# The epidemiology of chronic ankle instability with perceived ankle instability- a systematic review

**DOI:** 10.1186/s13047-021-00480-w

**Published:** 2021-05-28

**Authors:** Chiao-I Lin, Sanne Houtenbos, Yu-Hsien Lu, Frank Mayer, Pia-Maria Wippert

**Affiliations:** 1 Medical Sociology and Psychobiology, Department of Physical Activity and Health, University of Potsdam, Am Neuen Palais 10, House 12, 14469 Potsdam, Germany; 2 University Outpatient Clinic, Centre of Sports Medicine, University of Potsdam, Am Neuen Palais 10, House 12, Potsdam, Germany; 3 JC School of Public Health and Primary Care Faculty of Medicine, The Chinese University of Hong Kong, Shatin, NT, Hong Kong, China; 4 Faculty of Health Sciences Brandenburg, Joint Faculty of the University of Potsdam, the Brandenburg Medical School Theodor Fontane and the Brandenburg University of Technology Cottbus–Senftenberg, Am Neuen Palais 10, House 12, Potsdam, Germany

**Keywords:** Ankle sprain, Sports injury, Functional ankle instability

## Abstract

**Background:**

Chronic ankle instability, developing from ankle sprain, is one of the most common sports injuries. Besides it being an ankle issue, chronic ankle instability can also cause additional injuries. Investigating the epidemiology of chronic ankle instability is an essential step to develop an adequate injury prevention strategy. However, the epidemiology of chronic ankle instability remains unknown. Therefore, the purpose of this study was to investigate the epidemiology of chronic ankle instability through valid and reliable self-reported tools in active populations.

**Methods:**

An electronic search was performed on PubMed and Web of Science in July 2020. The inclusion criteria for articles were peer-reviewed, published between 2006 and 2020, using one of the valid and reliable tools to evaluate ankle instability, determining chronic ankle instability based on the criteria of the International Ankle Consortium, and including the outcome of epidemiology of chronic ankle instability. The risk of bias of the included studies was evaluated with an adapted tool for the sports injury review method.

**Results:**

After removing duplicated studies, 593 articles were screened for eligibility. Twenty full-texts were screened and finally nine studies were included, assessing 3804 participants in total. The participants were between 15 and 32 years old and represented soldiers, students, athletes and active individuals with a history of ankle sprain. The prevalence of chronic ankle instability was 25%, ranging between 7 and 53%. The prevalence of chronic ankle instability within participants with a history of ankle sprains was 46%, ranging between 9 and 76%. Five included studies identified chronic ankle instability based on the standard criteria, and four studies applied adapted exclusion criteria to conduct the study. Five out of nine included studies showed a low risk of bias.

**Conclusions:**

The prevalence of chronic ankle instability shows a wide range. This could be due to the different exclusion criteria, age, sports discipline, or other factors among the included studies. For future studies, standardized criteria to investigate the epidemiology of chronic ankle instability are required. The epidemiology of CAI should be prospective. Factors affecting the prevalence of chronic ankle instability should be investigated and clearly described.

## Background

Ankle sprain is one of the most common sports injuries in physically active individuals and causes a high financial burden on the healthcare system [1–3]. The incidence of ankle sprain from the US emergency departments in 2010 was 3.29 per 1000 person per year [4]. In an athletic population, a cohort of the sub-elite Australian football athletes showed an incidence of ankle sprain of 3.1 per 1000 athlete-exposures during the 2016 season [5]. In addition, 25 US collegiate sports presented an incidence of lateral ligament complex ankle sprain of 0.5 per 1000 athlete-exposures [1]. Regarding the substantial financial burden resulting from ankle sprain, Gribble et al. summarized that ankle sprains generated $6.2 billion in healthcare costs for US high-school athletes and €208 million in the Netherlands annually [3, 6]. In the US emergency department, $1029 per event of ankle sprain was charged [4].

Ankle sprain also predisposes athletes to recurrent ankle sprains and leads to residual symptoms [3, 7]. In soccer, basketball and volleyball, 61, 60 and 46% of the ankle sprain was recurrent ankle sprain [8]. Seventy-four percent of patients with an acute ankle sprain suffered from residual symptoms lasting 29 months after the initial ankle sprain, such as pain, perceived instability, weakness and swelling [9]. The International Ankle Consortium defines the pathology of residual symptoms after a significant ankle sprain as chronic ankle instability (CAI) [10]. The International Ankle Consortium characterized CAI as a condition in which an individual has a significant ankle sprain and/or experienced recurrent ankle sprain on the sprained ankle, and/or feels ankle instability, and/or experienced giving way at least twice in the past 6 months [10]. To determine the subjective ankle instability, three tools with a critical cutoff score are recommended by the International Ankle Consortium: The Ankle Instability Instrument (AII), The Cumberland Ankle Instability Tool (CAIT), and The Identification of Functional Ankle Instability (IdFAI) [10]. The criteria published by the International Ankle Consortium have been applied in research widely.

CAI is not only an ankle issue but also systematically affects other joints, causing further physical issues [11]. In the ankle structure, individuals with CAI show a decreased range of motion, secondary tissue injury, restricted osteokinematics and post-traumatic osteoarthritis [11]. CAI systematically impairs proprioception, balance, movement pattern, and invokes muscle weakness and altered H-reflex bilaterally [11]. CAI can cause further injuries, for example: recurrent ankle sprain, early development of osteoarthritis and increased loading on the anterior cruciate ligament [3, 12, 13]. Since CAI can lead to numerous negative consequences, it is important to develop a preventative strategy for this ankle problem. To develop a prevention strategy, the clarification of epidemiological data is essential [14].

To identify the prevalence of CAI in sporting populations, Attenborough and colleagues conducted a systematic review which defined CAI based on a CAI model published in 2011 [15] and reported the prevalence of the perceived ankle instability (28%), the recurrent ankle sprain (50%) and the persistent symptoms (30–45%) in basketball, soccer and volleyball [8]. However, the prevalence of CAI using the standardized criteria published in 2014 from the International Ankle Consortium has not been reported conclusively. Therefore, the purpose of this review was to identify the epidemiology of chronic ankle instability through valid and reliable self-reported tools in a physically active population.

## Methods

### Search strategy

The systematic search was performed in the online search engines PubMed and Web of Science in July 2020 using the keywords and MeSH terms (“ankle instab*” OR CAIT OR IDFAI OR AII) AND (prevalence OR frequency OR epidemiology). Articles published between 2006 and 2020 were screened, since the three tools (AII, CAIT and IdFAI) evaluating perceived ankle instability recommended by the International Ankle Consortium were published in 2006, 2006 and 2012 respectively.

### Inclusion and exclusion criteria

Studies that met the following criteria were included: (1) peer reviewed studies, (2) using one of the valid and reliable tools (AII, IdFAI, and CAIT) to evaluate chronic ankle instability, (3) determining chronic ankle instability based on the criteria of the International Ankle Consortium, (4) and the outcome represented the epidemiology of CAI. If the studies were not written in English, a review article or the full-text unavailable, they were excluded.

### Study selection and the data collection process

The study selection process was performed by two independent reviewers. After removing the duplicated articles, titles and abstracts of the articles were screened based on the pre-determined criteria. The remaining full-texts were reviewed for eligibility and either included or excluded for the current review. A third reviewer was consulted when the two authors could not reach agreements. Authors, published year, studied population, sample size, demographics, the criteria of determining CAI, inclusion criteria, exclusion criteria, and the epidemiological data of CAI were extracted.

### Risk of bias in individual studies

Two independent reviewers assessed the bias of included studies using an adapted risk assessment tool [16, 17]. There are seven items in the adapted bias assessment tool, including the definition of CAI, the study design, the description of participants’ demographics, the sampling method, the analysis rate of included data, the method of identifying CAI and the period of follow up (see Table 1). Each item was scored with a “Yes” or “No”, representing a high or low risk of bias respectively. The item was noted “No” if the information was not clear or the study did not meet the criteria of the specific item. When the score of risk of bias more than 75%, the risk of bias was considered low [27]. In case of different conclusions on scoring a certain item by the two reviewers, the discrepancies were discussed to reach an agreement.

**Table 1:** Risk of bias assessment

	Criterion	Schmitt et al. [18]	Donovan et al. [19]	Koshino et al. [20]	Holland et al. [21]	Doherty et al. [22]	Attenborough et al. [23]	Simon et al. [24]	Tanen et al. [25]	Kobayashi et al. [26]	% Studies with ‘yes’ response
1	That a clear definition of chronic ankle instability is clearly described	Yes	Yes	Yes	Yes	Yes	Yes	Yes	Yes	Yes	100%
2	Study design is cross-sectional or prospective	No	Yes	Yes	Yes	No	Yes	Yes	Yes	Yes	56%
3	Description of participants demographics are given	No	No	Yes	Yes	Yes	Yes	No	No	Yes	56%
4	Studies that conducted the random selection process or the studies that analyzed the entire target population receive	No	No	No	No	NA	No	Yes	No	No	13%
5	Prospective studies that collected the data of at least 80% of the participants included in the study. The cross-sectional and retrospective studies receive N/A for this criterion.	NA	NA	NA	NA	NA	NA	NA	NA	NA	NA
6	The injury diagnosis was conducted by health professionals or using valid and reliable tools	Yes	Yes	Yes	Yes	Yes	Yes	Yes	Yes	Yes	100%
7	The follow up period: For the prospective studies at least 6 months follow up, for retrospective studies up tp 12	NA	NA	NA	NA	Yes	NA	NA	NA	NA	NA
	Total score (%)	40%	60%	80%	80%	80%	80%	60%	40%	80%	

*NA* Not applicable

## Results

### Study selection

**Fig. 1:**
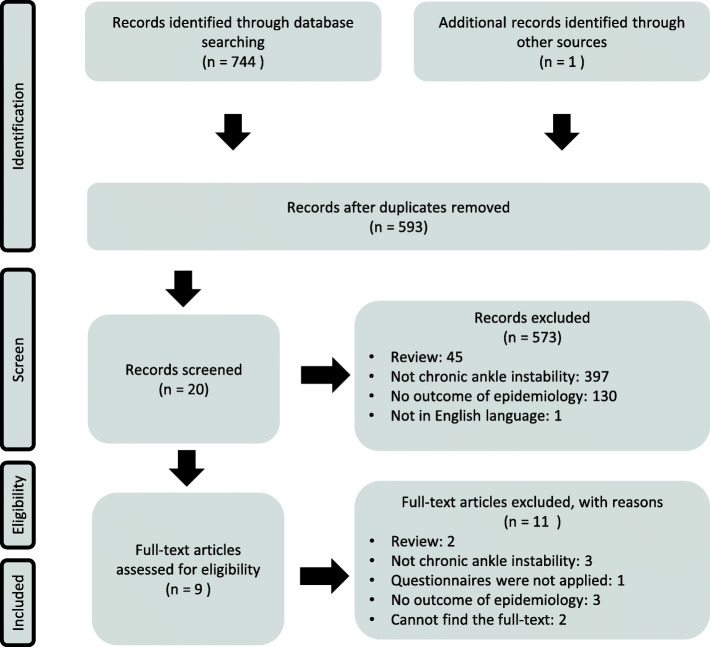
Flow chart of included and excluded studies

The conducted systematic searches resulted in a total of 744 studies (Fig. 1). After removing the 152 duplicates, the titles and abstracts of the 592 remaining articles were screened. Twenty articles entered the phase of full-text review and their references were screened for possible eligible articles. Although no other studies were found through this method in June 2020, one study published in September 2020 was included. Eventually, nine articles were included.

### Study characteristics and the results of individual studies

Table 2 provides a summary of the characteristics of the included studies. Three study types were included: seven cross-sectional studies [19–21, 23–26], one longitudinal descriptive study [18] and one cohort study [22]. The total sample size was 3804 participants. The sample size of each study ranged from 70 to 1238 participants.

**Table 2:** Summary of the included studies

Authors	Country	Study type	Mode of data collection	Study population	Defining CAI	Exclusion criteria	Prevalence of CAI, n(%)	Prevalence of CAI within participants with history of ankle sprains, n(%)	Participants with history of ankle sprain, n(%)
Schmitt et al. [18]	France	Descriptive longitudinal study	Questionnaires	French soldiers aged 32.2 (*N* = 1238) with a history of ankle sprain (*n* = 65)	Based on IAC using IdFAI	IAC	28 (2)	28/65 (43)	65 (5)
Donovan et al. [19]	USA	Cross-sectional study	Questionnaires	Athletes 8 from sports clubs and high Athletes in high schools in Wisconsin (*N* = 1002) Female (*n* = 505): 15.7 ± 1.7 years Male (*n* = 497): 15.6 ± 1.9 years	A history of ankle sprain and perceived ankle instability (evaluated using IdFAI)	Without history of injury	200 (20)	200/262 (76)	262 (26)
Koshino et al. [20]	Japan	Cross-sectional study	Questionnaires	Japanese Collegiate athletes with LAS (*N* = 470) CAI (*n* = 47): 20.0 ± 1.2 years, 1.70 ± 0.7 m, 65.5 ± 11.5 kg Coper (*n* = 20): 20.5 ± 1.4 years, 1.68 ± 0.06 m, 63.8 ± 10.3 kg	Based on IAC using CAIT	Research criteria: IAC Clinical criteria: IAC without exclusion criteria	47 (10) 93 (20)	47/212 (22) 93/212 (44)	212 (45)
Holland et al. [21]	USA	Cross-sectional study	Questionnaires	Students in western North Carolina(*N* = 201) Uninjured (*n* = 86): 16.0 years, 1.69 m, 62.0 kg Coper (*n* = 16): 15.38 years, 1.66 m, 65.2 kg Potentially unstable (*n* = 40): 15.6 years, 1.65 m, 60.9 kg Unstable (*n* = 59): 15.68 years, 1.68 m, 62.5 kg	Based on IAC using IdFAI	Missing data	59 (29)	59/115 (51)	115 (57)
Doherty et al. [22]	Ireland	Cohort study	Questionnaires	Physical active individuals with LAS (*N* = 70) CAI (*n* = 28): 23.2 years, 75.5 kg, 1.72 m LAS coper (*n* = 42): 22.7 years, 73.4 kg, 1.73 m	Based on IAC using CAIT	IAC	NA	28/70 (40)	NA
Attenborough et al. [23]	Australia	Cross-sectional study	Questionnaires Ankle joint laxity	Female netball players in Sydney(*N* = 96) Club (n = 42): 24.1 ± 7.9 years, 1.67 ± 0.05 m, 68.5 ± 15.9 kg Inter-district (*n* = 54): 19.4 ± 3.5 years, 1.73 ± 0.06 m, 72.0 ± 12.7 kg	Previous ankle sprain + Recurrent ankle sprain, perceived ankle instability (CAIT), mechanical ankle instability	A lower limb injury in the 6 months prior to testing a history of ankle surgery or ankle fracture all previous ankle sprains occurred a minimum of 6 months prior	44 (46)	44/69 (64)	69 (72)
Simon et al. [24]	USA	Exploratory study	Questionnaires	Dancer (*N* = 77) 19.61 ± 2.53 years, dance experience: 13.61 ± 3.16 years	perceived ankle instability (IdFAI)	A history of fracture or surgery in the lower extremities.	41 (53)	41/54 (76)	54 (70)
Tanen et al. [25]	USA	Descriptive epidemiological survey	Questionnaires	Athletes (*N* = 512) Collegiate athletes (*n* = 316) 19.6 ± 1.2 years high school athletes (*n* = 196) 15.9 ± 1.2 years	Perceived ankle instability (CAIT and AII)	A history of an ankle fracture, ankle surgery, neurological disorder such as, Parkinson’s disease, amyotrophic lateral sclerosis, or multiple sclerosis, or failed to completely answer the questionnaires.	120 (23)	120/391 (31)	391 (76)
Kobayashi et al. [26]	Japan	Cross-sectional study	Questionnaires	Female athletes (*N* = 138) Aged 18–23 21.8 ± 0.4 years, 1.66 m, 57.0 kg	Based on IAC using CAIT	IAC	10 (7)	10/106 (9)	106 (77)

*IAC* The International Ankle Consortium, *CAIT* The Cumberland Ankle Instability Tool, *AII* The ankle Instability Instrument, *IdFAI* The Identification of functional Ankle Instability, *NA* not applicable

The summarized prevalence of CAI was 25% (ranging from 7 to 53%). The data from one study was not integrated into the overall result because the study applied the prevalence of 65 included participants who sought medical care to estimate the prevalence for the whole population (*N* = 1238) [18]. Forty-six percent of participants with a history of ankle sprains were diagnosed with CAI (ranging from 9 to 76%).

The age of the participants among the different study populations ranged from 15 to 32 years. The number of participants aged younger than 18 years was 1399 [21, 22, 25], for adult participants (18–24 years) it was 1167 [19, 20, 23–26] and the number of participants for the military population (average age was 32 years) was 1238 [18]. The prevalence of CAI in participants aged younger than 18 years, aged between 18 and 24 years and aged over 25 years were 26% (320/1399) [19, 21, 25], 25% (237/959) [20, 23–25], and 2% (28/1238) [18] respectively. The prevalence of CAI in participants with a history of ankle sprain in each age category was 63, 36 and 43%.

The study population consists of military personnel [18], athletes [19, 20, 23, 25, 26], dancers [24], and physically active individuals [21, 22]. The prevalence of CAI within each sport is shown in Table 3.

**Table 3:** Prevalence of chronic ankle instability in different sports

Sport	Total	CAI		Having history of ankle sprain	
	n	n	%	n	%
Netball [23]	96	44	46%	69	72%
Dance [24, 26]	99	45	45%	69	70%
Aquatics [25]	50	16	32%	28	56%
Basketball [20, 25, 26]	105	32	30%	85	81%
Volleyball [20, 25, 26]	79	24	30%	53	67%
Rowing/Crew [25]	10	3	30%	8	80%
Field Hockey [25]	11	3	27%	7	64%
Wrestling [25]	23	6	26%	16	70%
Rugby [20]	35	9	26%	22	63%
Acrobatics [25]	35	9	26%	22	6%
Baseball [25]	38	9	24%	34	89%
Judo [20]	18	4	22%	6	33%
Running [25]	66	14	21%	29	44%
Soccer [20, 25, 26]	108	22	20%	77	71%
Gymnastics [20]	15	3	20%	9	60%
Handball [20]	5	1	20%	2	40%
Golf [25]	11	2	18%	5	45%
Lacrosse [20]	60	9	15%	23	38%
Ice hockey [20]	31	3	10%	11	35%
Swimming [26]	11	1	9%	6	55%
Tennis [20, 25]	55	4	7%	20	36%
Badminton [20]	14	1	7%	7	50%
Track and field [20, 26]	63	5	8%	23	37%
Table tennis [20]	55	2	4%	12	22%
Total	1093	99	25%	643	47%

*CAI* chronic ankle instability

The definitions of CAI in the included studies were homogeneous, but the exclusion criteria diverse. Four articles applied the inclusion and exclusion criteria from the International Ankle Consortium to define the presence of CAI (2.3–40%) [18, 20, 22, 26]. Two studies only applied inclusion criteria from the International Ankle Consortium (10 and 49%) [20, 21]. One study identified CAI by the history of a significant ankle sprain, mechanical ankle instability and the perceived ankle instability (46%) [23]. Three studies used the history of ankle sprains and perceived ankle instability to identify CAI (20–53%) [19, 24, 25].

### Risk of bias across studies

The results of the critical appraisal are displayed in Table 1. Two studies scored less than 60% [18, 25], two studies scored 60% [19, 24] and five studies scored ≥80% [20–23, 26] on the bias assessment tool. Seven included studies showed clear criteria to define CAI by the same or similar to the criteria from the International Ankle Consortium [18, 20, 22, 23, 25, 26]. The criteria for the two studies were unclear but clarified after contacting the corresponding authors [19, 21]. In regards to the study design, five studies were cross-sectional studies, and the other studies were a longitudinal descriptive study [18], a cohort study [22], an exploratory study [24] and a descriptive epidemiological survey [25]. For the demographics of the participants, four studies only provided age [18, 19, 21, 24]. None of the included studies applied random selection to sampling. One study defined the target population clearly at a university and analyzed all data they received [24]. Three included studies clearly defined the target population but did not analyze the entire target population [18, 19, 21]. Four studies did not either define the target population precisely or report the sampling process [20, 23, 25, 26]. Only one study followed up participants with first-time ankle sprain for 12 months [22]. All studies applied standard tools to evaluate ankle instability.

## Discussion

The current review included nine studies and the results showed that the prevalence of CAI was 25%, ranging from 7 to 53% and the prevalence of CAI within participants with a history of ankle sprains was 46%, ranging from 9 to 76%. Five out of nine included studies had a low risk of bias.

The prevalence of CAI from eight included studies ranged from 7 to 53%. The results from one study were not integrated because of the high risk of bias [18]. The wide range of prevalence may be caused by the varying research methods, different characteristics of participants and other factors. In regards to varying research methods, the included studies applied different exclusion criteria to investigate the prevalence of CAI (Table 2), although the International Ankle Consortium published standard inclusion and exclusion criteria aimed at controlled research [10]. Based on the criteria participants with CAI and other conditions (e.g. history of a fracture and surgeries in lower extremities) will be excluded because the conditions confound the CAI symptoms (e.g. giving way and perceived ankle instability) [10]. In this case, the prevalence might be underestimated. Yet, if studies defined CAI based on inclusion criteria only (a history of one or more significant ankle sprain and experienced “giving way” and/or recurrent sprain and/or “feelings of instability”) and do not exclude the participants without CAI but with the conditions in the exclusion criteria, the prevalence might be overestimated.

Most of the included studies investigated CAI using a questionnaire and excluded participants with the conditions confounding the presence of ankle instability (Table 2). Without history-taking or physical examination, it is difficult to differentiate CAI from other possible conditions in the exclusion criteria. Koshino et al. found that by applying both inclusion and exclusion criteria from the International Ankle Consortium to determine the prevalence of chronic ankle instability the prevalence was 10.0% [20]. Yet, by only applying the inclusion criteria the prevalence was doubled (19.8%) [20]. Thus, a comprehensive method should be established for the research of CAI epidemiology which can differentiate between CAI and other issues confounding the symptoms of CAI.

In addition, different characteristics of participants, for example age and population, also vary the prevalence of CAI. Regarding to age, the samples from the included studies come with a wide range of ages (15–32 years). A previous study showed that a younger age is one of the risk factors for recurrent ankle sprain, which in turn is one of the risk factors of CAI [28]. Tanen et al. showed that high school athletes had a higher prevalence of CAI compared to collegiate athletes (31 and 19% respectively) [25]. In the current review, the prevalence of CAI with a history of ankle sprain seems higher in participants aged younger than 18 years (63%) compared to those aged between 18 and 25 years (36%). The included studies represent a wide range of ages that may be responsible for the wide range of prevalence.

Furthermore, different sports disciplines show a varying prevalence of ankle sprain and CAI. Doherty et al. found that indoor/court sports showed the highest prevalence and incidence of ankle sprain (7 ankle sprains per 1000 athletic exposure [95% CI 6.8–7.2], 12.17% [95% CI 12.01–12.33]) among water/ice sports (3.7/1000 athletic exposure [95% CI 3.3–4.17], 4.36%[95% CI 3.92–4.79]), filed-based sports (1.0/1000 athletic exposure [95% CI 0.95–1.05], 11.3% [95% CI 11.15–11.44]) and outdoor pursuits sports (0.88/1000 athletic exposure [95% CI 0.73–1.02], 11.65% [95% CI 11.33–11.97]) [2]. Roos et al. discovered that basketball had the highest rate of lateral ankle sprain from 25 sports [1]. Regarding the recurrent ankle sprain, athletes showed the highest rate of recurrent ankle sprain in basketball, women’s outdoor track and women’s hockey from 25 sports disciplines [1]. Besides, a systematic review revealed that soccer, basketball and volleyball reported the highest rate of recurrent ankle sprain, and track and field showed the most participants perceived ankle instability with a history of ankle sprain [8]. Similarly, Koshino et al. found that athletes who play basketball (63%, 14/22), volleyball (42%, 11/26) or soccer (37%, 15/40) had a high rate of CAI [20]. In the included studies from the current review, netball, dance and aquatics show the highest prevalence of CAI followed by basketball, volleyball and rowing/crew (Table 3).

Additionally, the investigated populations and the sample size of each sport discipline varied among the included studies. For example, the sample size in the various included sports ranged between five and 163 participants. There were only 10 to 20 participants overall in the categories of swimming, golf, gymnastics, field hockey and rowing/crew, and there were 96 to 108 participants in netball, dance, basketball and soccer (Table 3). It is difficult to generalize the result due to the varying age, sports disciplines and wide range of sample sizes in each sports discipline of the participating populations. Therefore, clear description of the factors (for example, age [28] and sports discipline [2]) in epidemiological study of CAI can facilitate a comprehensive understanding of CAI prevalence.

Some articles investigating the epidemiology of CAI were excluded CAI due to the mismatched definition from the current review. Two systematic reviews defined CAI as self-reported perceived ankle instability, mechanical instability, repetitive ankle sprain and persisting symptoms lasting over 6 months after an acute ankle sprain and surveyed the epidemiology of CAI in sporting populations and children [8, 29]. In sporting populations, the recurrent ankle sprain (61%) was the most prevalent in soccer athletes and the highest rate of perceived ankle instability (41%) was in track and field athletes with a history of ankle sprain [8]. Children with a history of ankle sprain and perceived ankle instability/recurrent ankle sprain was 22–71% [29]. The other excluded study defined CAI as recurrent ankle sprain or ankle functional impairment or mechanical ankle instability or residual symptoms after 1 year of ankle sprain and found the prevalence of CAI was 1% in a 17-year-old general population (*N* = 829,791) who were recruited into mandatory military service [30]. Again, with the heterogeneous population and definition of CAI, the rate of CAI can range from 1 to 71% which is a wider range than in the current results (7 to 53%).

Regarding other factors affecting the prevalence of CAI, accessibility of rehabilitation can affect the development of CAI. Exercise therapy showed moderate evidence to treat/prevent recurrent ankle sprain [31]. For instance, proprioception training reduces 36% of the risk in recurrent ankle sprain in the participants with a history of ankle sprain [32]. Balance training can also improve the perceived ankle instability of the patients with CAI [33]. However, Hubbard-Turner discovered that 64% (112/175) of the participants did not seek medical care after lateral ankle sprain injuries, and within the 36% (63/175) who seek treatment, only 10% (6/63) of them performed balance training [34]. Similarly, Schmitt et al. found that 47.6% of participants did not receive physiotherapy after the first ankle sprain [18] and Tanen et al. found that 45% of the investigated athletes did not seek medical care [25]. Doherty et al. showed that 40% of the participants who did not seek exercise/physical therapy developed CAI, whereas 60% of the participants who received rehabilitation did not develop CAI, although there was no significant association between rehabilitation and the development of CAI [35]. The availability of exercise/physical therapy may differ from areas and institutions. However, most of the included studies did not present the history of rehabilitation for ankle sprains, which may confound the results [10].

Additionally, there are some other influencing factors that have been discussed in previous studies, for example, body size, gender and competition level. Unfortunately, the evidence is not conclusive. For body size, one cross-sectional study found that participants’ body mass index and height are associated with the presence of mechanical and functional ankle instability in a general population (*N* = 829,791) [30]. However, a prospective study found that body mass index is not associated with the recovery of ankle function 6 months after an acute ankle sprain [36]. The difference in the prevalence of CAI between genders also remains unclear. Regarding gender, one of the included studies found that female athletes showed a higher prevalence of CAI than male athletes (32% vs. 17% respectively, *p* < 0.05) [25]. In addition, Donovan et al. found that the prevalence for boys was 23.5% and for girls it was 26.2% [19]. In contrast, Hershkovich et al. found that men had a 2.33 fold-greater incidence of CAI than women (1.1% vs. 0.7%, *N* = 829,791) [30].

Competition levels may play an essential role on the prevalence of CAI, but the direction is controversial in the current evidence. Tanen et al. found that the prevalence of CAI was higher in the athletes in a lower competitive level (high school athletes) than that in a higher competitive level (collegiate athletes) [25]. Although Attenborough et al. further showed that athletes in the lower competition levels (club athletes) had a higher prevalence of CAI than that in the higher competition levels (inter-district athletes), the average age for club and inter-district athletes being 19 and 24 years [23]. It is not clear if the difference in the prevalence between these two populations were from the age, the competitive level, or both. In future studies, body size, gender, competitive level and history of rehabilitation after an acute ankle sprain should be identified to understand their effects on the prevalence of CAI and the above factors should be clearly described to depict the characteristics of the surveyed cohort.

### Risk of bias

The criteria to define CAI were applied in each study. Although all studies define CAI based on the standard criteria of the International Ankle Consortium, the inclusion and exclusion criteria were distinct among the included studies. This causes a misestimating of the prevalence of CAI. As mentioned in the previous paragraph, the standard from the International Ankle Consortium is for controlled research, which excludes the participants with other issues confounding identification of CAI. The participants with other conditions (history of a fracture or surgeries or acute injury in previous 3 months) and CAI cannot be clarified. This will definitely affect the results of the CAI prevalence. Therefore, to establish the standard criteria is a prerequisite for conducting epidemiological studies.

In regards to the study design, to investigate the epidemiology of chronic injuries, Bahr suggested applying a prospective study design with continuous or serial measurements [37]. However, none of the included studies applied the prospective study design. The prevalence would fluctuate among different game seasons. Therefore, the data from each study can only represent the prevalence in a certain period. Future studies should be prospective designed to measure the symptoms of CAI at regular intervals and to portray the presence of CAI among whole seasons.

Participants’ characteristics were missing in four included studies [18, 19, 24, 25]. Height, body mass index and age are associated with CAI [30]. Without the characteristics of the sample, it is difficult to generalize the data. Seven included studies did not analyze the whole target population or clearly define the target population [18–21, 23, 25, 26]. This could affect the representation and the generalization of the data.

There were some limitations in the current review. First of all, only nine studies were included. The prevalence might not be representative because of the small sample size. In addition, the included studies were heterogeneous. The surveyed population, countries, competitive level and sports were varying. Three studies presented the prevalence of CAI in different sports [20, 25, 26]. Furthermore, the criteria to define CAI were different among the included studies. A clear standard to define CAI in future epidemiological studies should be defined. Finally, it is not clear if the pre-existing ankle instability affects the development of CAI after a significant ankle sprain. Some individuals have perceived ankle instability or giving way without a history of ankle sprain. Do the individuals have CAI because of the pre-existing instability, or do they really develop CAI after a significant ankle sprain?

## Conclusion

The prevalence of chronic ankle instability in the active population was 25%, ranging between 7 and 53% in different populations. The prevalence of chronic ankle instability within the participants with a history of an ankle sprain was 46%, ranging from 9 to 76%. The wide range of the prevalence was mainly caused by exclusion criteria, age, sports discipline, and other factors. In order to obtain comprehensive epidemiological information about CAI, first of all, prospective studies should be conducted to the symptoms of CAI with valid and reliable tools at regular intervals [37]. To report the injury risk of CAI, prevalence should be used, because athletes with CAI still participate in practice and competitions [37]. In addition, the thorough method to well identify the participants with CAI and other lower limbs condition should be developed. Finally, the risk factors of ankle sprain or CAI including age and sports discipline should be clearly reported to depict the surveyed population. Factors which remains unclear of ankle sprain/CAI (e.g. gender, body size and history of rehabilitation) should be clarified and described in further epidemiology studies of CAI.

## Data Availability

Not applicable.

## Abbreviations

CAI: Chronic ankle instability
AII: The Ankle Instability Instrument
CAIT: The Cumberland Ankle Instability Tool
IdFAI: The Identification of Functional Ankle Instability
IAC: The International Ankle Consortium

## References

[1] Roos KG, Kerr ZY, Mauntel TC, Djoko A, Dompier TP, Wikstrom EA (2016). The epidemiology of lateral ligament complex ankle sprains in National Collegiate Athletic Association Sports. Am J Sports Med.

[2] Doherty C, Delahunt E, Caulfield B, Hertel J, Ryan J, Bleakley C (2014). The incidence and prevalence of ankle sprain injury: a systematic review and meta-analysis of prospective epidemiological studies. Sports Med.

[3] Gribble PA, Bleakley CM, Caulfield BM, Docherty CL, Fourchet F, Fong DT (2016). Evidence review for the 2016 international ankle consortium consensus statement on the prevalence, impact and long-term consequences of lateral ankle sprains. Br J Sports Med.

[4] Shah S, Thomas AC, Noone JM, Blanchette CM, Wikstrom EA (2016). Incidence and cost of ankle sprains in United States emergency departments. Sports Health.

[5] Whalan M, Lovell R, McCunn R, Sampson JA (2019). The incidence and burden of time loss injury in Australian men’s sub-elite football (soccer): a single season prospective cohort study. J Sci Med Sport.

[6] Knowles SB, Marshall SW, Miller T, Spicer R, Bowling JM, Loomis D, Millikan RW, Yang J, Mueller FO (2007). Cost of injuries from a prospective cohort study of North Carolina high school athletes. Inj Prev.

[7] Owoeye OBA, Palacios-Derflingher LM, Emery CA (2018). Prevention of ankle sprain injuries in youth soccer and basketball: effectiveness of a neuromuscular training program and examining risk factors. Clin J Sport Med.

[8] Attenborough AS, Hiller CE, Smith RM, Stuelcken M, Greene A, Sinclair PJ (2014). Chronic ankle instability in sporting populations. Sports Med.

[9] Anandacoomarasamy A, Barnsley L (2005). Long term outcomes of inversion ankle injuries. Br J Sports Med.

[10] Gribble PA, Delahunt E, Bleakley C, Caulfield B, Docherty C, Fourchet F, et al. Selection criteria for patients with chronic ankle instability in controlled research: a position statement of the International Ankle Consortium. Br J Sports Med. 2014;48(13). 10.1136/bjsports-2013-093175. 10.1136/bjsports-2013-093175 24255768

[11] Hertel J, Corbett RO (2019). An updated model of chronic ankle instability. J Athl Train.

[12] Wikstrom EA, Song K, Tennant JN, Dederer KM, Paranjape C, Pietrosimone B (2019). T1ρ MRI of the talar articular cartilage is increased in those with chronic ankle instability. Osteoarthr Cartil.

[13] Terada M, Pietrosimone B, Gribble PA (2014). Individuals with chronic ankle instability exhibit altered landing knee kinematics: potential link with the mechanism of loading for the anterior cruciate ligament. Clin Biomech.

[14] Bahr R, Krosshaug T (2005). Understanding injury mechanisms: a key component of preventing injuries in sport. Br J Sports Med.

[15] Hiller CE, Kilbreath SL, Refshauge KM (2011). Chronic ankle instability: evolution of the model. J Athl Train.

[16] Lopes AD, Hespanhol Júnior LC, Yeung SS, Costa LOP (2012). What are the main running-related musculoskeletal injuries? a systematic review. Sports Med (Auckland, NZ).

[17] Keogh JWL, Winwood PW (2017). The epidemiology of injuries across the weight-training sports. Sports Med.

[18] Schmitt M, Marchi J, Jouvion A, Trappier T, Reyes-Rivet L, De Brier G (2020). Prevalence of chronic ankle instability in French paratroopers. Mil Med.

[19] Donovan L, Hetzel S, Laufenberg CR, McGuine TA (2020). Prevalence and Impact of Chronic Ankle Instability in Adolescent Athletes. Orthop J Sports Med.

[20] Koshino Y, Samukawa M, Murata H, Osuka S, Kasahara S, Yamanaka M, Tohyama H (2020). Prevalence and characteristics of chronic ankle instability and copers identified by the criteria for research and clinical practice in collegiate athletes. Phys Ther Sport.

[21] Holland B, Needle AR, Battista RA, West ST, Christiana RW (2019). Physical activity levels among rural adolescents with a history of ankle sprain and chronic ankle instability. PLoS One.

[22] Doherty C, Bleakley C, Hertel J, Caulfield B, Ryan J, Delahunt E (2018). Clinical tests have limited predictive value for chronic ankle instability when conducted in the acute phase of a first-time lateral ankle sprain injury. Arch Phys Med Rehabil.

[23] Attenborough AS, Sinclair PJ, Sharp T, Greene A, Stuelcken M, Smith RM, Hiller CE (2016). A snapshot of chronic ankle instability in a cohort of netball players. J Sci Med Sport.

[24] Simon J, Hall E, Docherty C (2014). Prevalence of chronic ankle instability and associated symptoms in university dance majors: an exploratory study. J Dance Med Sci.

[25] Tanen L, Docherty CL, Van Der Pol B, Simon J, Schrader J (2013). Prevalence of chronic ankle instability in high school and division I athletes. Foot Ankle Spec.

[26] Kobayashi T, Takabayashi T, Kudo S, Edama M (2020). The prevalence of chronic ankle instability and its relationship to foot arch characteristics in female collegiate athletes. Phys Ther Sport.

[27] Nauta J, Martin-Diener E, Martin BW, van Mechelen W, Verhagen E (2015). Injury risk during different physical activity Behaviours in children: a systematic review with Bias assessment. Sports Med.

[28] Pourkazemi F, Hiller CE, Raymond J, Black D, Nightingale EJ, Refshauge KM (2018). Predictors of recurrent sprains after an index lateral ankle sprain: a longitudinal study. Physiotherapy..

[29] Mandarakas M, Pourkazemi F, Sman A, Burns J, Hiller CE (2014). Systematic review of chronic ankle instability in children. J Foot Ankle Res.

[30] Hershkovich O, Tenenbaum S, Gordon B, Bruck N, Thein R, Derazne E, Tzur D, Shamiss A, Afek A (2015). A large-scale study on epidemiology and risk factors for chronic ankle instability in young adults. J Foot Ankle Surg.

[31] Doherty C, Bleakley C, Delahunt E, Holden S (2017). Treatment and prevention of acute and recurrent ankle sprain: an overview of systematic reviews with meta-analysis. Br J Sports Med.

[32] Rivera MJ, Winkelmann ZK, Powden CJ, Games KE (2017). Proprioceptive training for the prevention of ankle sprains: an evidence-based review. J Athl Train.

[33] Rozzi SL, Lephart SM, Sterner R, Kuligowski L (1999). Balance training for persons with functionally unstable ankles. J Orthop Sports Phys Ther.

[34] Hubbard-Turner T (2019). Lack of medical treatment from a medical professional after an ankle sprain. J Athl Train.

[35] Doherty C, Bleakley C, Hertel J, Caulfield B, Ryan J, Delahunt E (2016). Recovery from a first-time lateral ankle sprain and the predictors of chronic ankle instability: a prospective cohort analysis. Am J Sports Med.

[36] Bielska IA, Brison R, Brouwer B, Janssen I, Johnson AP, Day AG, Pickett W (2019). Is recovery from ankle sprains negatively affected by obesity?. Ann Phys Rehabil Med.

[37] Bahr R (2009). No injuries, but plenty of pain? On the methodology for recording overuse symptoms in sports. Brit J Sport Med.

